# Job satisfaction of public and private primary care physicians in Malaysia: analysis of findings from QUALICO-PC

**DOI:** 10.1186/s12960-019-0410-4

**Published:** 2019-11-04

**Authors:** N. Ab Rahman, M. Husin, K. Dahian, K. Mohamad Noh, R. Atun, S. Sivasampu

**Affiliations:** 1 Institute for Clinical Research, National Institutes of Health (NIH), Ministry of Health Malaysia, Block B4, No. 1, Jalan Setia Murni U13/52, 40170 Shah Alam, Selangor Malaysia; 2 Clinical Research Centre, Sibu Hospital, Ministry of Health Malaysia, Sibu, Sarawak Malaysia; 3 Faculty of Medicine, Cyberjaya University College of Medical Sciences, Cyberjaya, Selangor Malaysia; 4 Harvard T.H. Chan School of Public Health, Harvard University, Boston, MA United States of America

**Keywords:** Job satisfaction, Public sector, Private sector, Primary care, Health professionals

## Abstract

**Background:**

Job satisfaction of doctors is an important factor determining quality and performance of a health system. The aim of this study was to assess job satisfaction among doctors of the public and private primary care clinics in Malaysia and evaluate factors that could influence the job satisfaction rating.

**Methods:**

This study was part of the Quality and Costs of Primary Care (QUALICOPC) Malaysia, a cross-sectional survey conducted between August 2015 and June 2016 in Malaysia. Data was collected from doctors recruited from public and private primary care clinics using a standardised questionnaire. Comparisons were made between doctors working in public and private clinics, and logistic regression analysis was used to determine factors influencing the likelihood of job satisfaction outcomes.

**Results:**

A total of 221 doctors from the public and 239 doctors from the private sector completed the questionnaire. Compared to private doctors, a higher proportion of public doctors felt they were being overloaded with the administrative task (59.7% vs 36.0%) and part of the work does not make sense (33.9% vs 18.4%). Only 62.9% of public doctors felt that there was a good balance between effort and reward while a significantly higher proportion (85.8%) of private doctors reported the same. Over 80% of doctors in both sectors indicated continued interest in their job and agreed that being a doctor is a well-respected job. Logistic regression analysis showed public-private sector and practice location (urban-rural) to be significantly associated with work satisfaction outcomes.

**Conclusion:**

A higher proportion of public doctors experienced pressure from administrative tasks and felt that part of their work does not make sense than their colleague in the private sector. At the same time, the majority of private doctors reported positive outcome on effort-and-reward balance compared to only one third of public doctors. The finding suggests that decreasing administrative workload and enhancing work-based supports might be the most effective ways to improve job satisfaction of primary care doctors because these are some of the main aspects of the job that doctors, especially in public clinics, are most unhappy with.

## Introduction

A strong primary health care system is widely regarded as one of the best approaches for the delivery of cost-effective health services to achieve and maintain universal health coverage (UHC) [1]. Attaining the main goal of offering the highest quality of health services and best health outcomes possible depends on the availability and accessibility of health workers as well as committed and well-performing workforce [2]. It has become increasingly important given the current shift in the global disease burden, i.e. the rapid rise of non-communicable diseases (NCDs), persistent problems with communicable diseases, and an ageing population that will bring an influx of people into the primary care system [3, 4]. This creates an increase in demand for primary care services while the issue of workforce shortage prevails. Therefore, keeping the primary care physicians satisfied and motivated is essential as it helps the entire health system to work smoothly.

Malaysia, an upper middle-income country, has a dichotomous healthcare system spread between the government-subsidised public sector and fee-for-service private sector. The public tier delivers comprehensive, affordable care to the citizen through a system of community-based primary healthcare facilities linked to secondary and tertiary hospitals offering more specialised in- and outpatient services. The private tier parallels in many ways the public system, tending to service wealthier elements of the society who can afford the out-of-pocket payment of higher fees [5, 6]. Although tangible accessibility is guaranteed through the mixed public-private health system, the effective workforce in the Malaysian healthcare is considered low compared to its healthcare needs and human asset [7, 8]. Currently, Malaysia has 1.5 doctors per 1000 population; although higher than neighbouring Southeast Asian countries like Thailand (0.8) and Vietnam (0.8), the number is lower than the levels observed in its closest neighbour Singapore (2.3) and countries such as Japan (2.4) and Australia (3.5) [7]. For primary care, per capita density of primary care physicians in Malaysia is 1.5 per 1000 population in the urban areas and 1.1 per 1000 population in the rural areas [9]. Besides, there is considerable variation across public and private sectors in terms of organisation, financing, governance, delivery of services, and patient and provider profiles [10, 11]. Private clinics are concentrated in urban, affluent areas and cities whereas public clinics’ coverage is wider including those in rural areas. Although there are five times more primary care clinics in the private sector compared to the public sector, higher patient visits were recorded in public clinics [9], resulting in an overload of patients and clogging up the clinics’ capacity. Retention of primary care physicians remains a challenge, particularly for the public sector [9, 12]. The difference in the public and private sector may create potential disparities in job-related attitudes among physicians. For instance, in a study by Aidalina et al., dissatisfaction with the work condition was cited as the main reason for physicians to migrate from the public to the private sector [13].

Assessment of physicians’ job satisfaction is one of the approaches to look into existing healthcare situation and possible problems. Physicians’ well-being and job satisfaction impact health service quality; thus, it is regarded as one of the outcomes of healthcare and has become an increasingly important subject during the course of health reforms [14, 15]. Job satisfaction of physician impacts productivity, aspects of quality of care, and patient satisfaction with care [16, 17]. More importantly, job satisfaction has been identified as an important determinant of physician turnover and retention [18–20]. Several local studies on job satisfaction of healthcare workers mostly focused on the public sector [21–23] while only a few specifically address primary care doctors [23, 24]. Although higher job satisfaction level among physicians who work in the private sector compared to their counterpart in the public sector has been reported in other countries [25, 26], it is not known whether such differences exist among primary care physicians in Malaysia. Survey results in other countries showed that physicians were dissatisfied with aspects related to working hours and administrative tasks while rewards, recognition, and remuneration also influenced their work satisfaction [27–29]. Prior studies also revealed that physicians’ job satisfaction is health system dependent while a number of factors such as individual, organisational, and work factors may also be associated with the degree of job satisfaction [16, 30–32]. However, most of these studies have come out of high-income country settings, and it might not be applicable to low- and middle-income country health care systems because the nature of the systems is different. By understanding issues faced by primary care physicians, it is hoped that (a) a process can be developed to address them and (b) the middle-income country lessons of Malaysia may support regional neighbours and other LMIC with similar issues.

The present study aims to address the abovementioned gaps. Malaysia’s participation in the large international Quality and Costs of Primary Care (QUALICOPC) study during the period 2015–2016 reflects on the current state of primary care in Malaysia which also allows for comparison with other countries [4]. Within the QUALICOPC framework, we had the opportunity to measure job satisfaction of primary care physicians in Malaysia according to their perceptions of several work aspects. This could also reflect challenges faced by primary care physicians in Malaysia that have not been formally investigated. In this study, we aimed to compare levels of job satisfaction between physicians working in public and private primary care clinics in Malaysia. Additionally, we looked at physician, organisational, and job characteristics likely to affect the job satisfaction rating.

## Methods

### Design

QUALICOPC is a multi-country study that evaluates measures of quality, costs, and equity in primary health care across countries [33, 34]. This cross-sectional survey uses a set of four questionnaires: the General Practitioner (GP) questionnaire, the Patient Experience Questionnaire, the Patient Value questionnaire, and the Practice (Fieldworker) questionnaire [34]. Using these standard international instruments, the study was conducted in Malaysia between August 2015 and June 2016. The general design and method have been described in detail elsewhere [35]. In summary, public and private primary care clinics from five states in Malaysia were selected through stratified random sampling. A minimum sample size of 220 clinics for each country has been pre-determined for all countries involved in the QUALICOPC study [33]. For Malaysia, we targeted 220 clinics from the public sector and another 220 clinics from the private sector. The questionnaires were adapted for local context, and to ensure it captures the desired constructs, the adaptations were done as such that the questionnaires remained as close as possible to the original. For content validity, the questionnaire was reviewed by a committee consisting of two family medicine specialists and three researchers. Terms and response categories not commonly used in the local setting were identified and changed.

In every clinic, one doctor and 10 of the doctor’s patients were asked to complete the questionnaires through an interview with trained fieldworkers. Nine patients filled in the Patient Experience questionnaire, one patient filled in the Patient Value questionnaire, and the fieldworkers filled in the Practice questionnaire about the facility. Doctors were asked to fill in the GP questionnaire which comprised 60 questions concerning structural aspects of the primary care practice, workload, care processes, and their demographic details [34]. The GP questionnaire was in English (Additional file 1). All responses were anonymous, and participation in the study was completely voluntary.

### Job satisfaction variables

Six questions from the GP questionnaire were used as a proxy to job satisfaction measures: “I feel that some part of my work does not make any sense”, “My work still interest me as much as it ever did”, “My work is overloaded with unnecessary administrative details”, “I have too much stress in my current job”, “Being a doctor is a well-respected job”, “In my work there is a good balance between effort and reward”. For each question, doctors were asked to indicate whether they agreed with the statement by selecting one of the following responses: “strongly agree”, “agree”, “disagree”, or “strongly disagree”. Outcomes are presented in (i) numerical and (ii) binary response:

(i) The responses were coded from 1 to 4, with higher scores reflecting higher satisfaction (1 = low job satisfaction and 4 = high job satisfaction). Responses from the following questions were reverse coded to keep the scale in the same direction: “work still interesting”, “well-respected”, and “balance”. For example, a high score on “balance” reflects a higher level of agreement on the statement, which indicates high satisfaction.
(ii) The responses were dichotomously coded as a binary variable with 0 reflecting “disagree” (combing strongly disagree and disagree) and 1 reflecting “agree” (combining strongly agree and agree).

### Independent variables

Independent variables to be included in the analysis were identified from the GP questionnaires based on relevancy to local practice setting and factors that were previously identified in earlier studies [16, 30, 36]. Physician variables included age, sex, and birth country. Job or workload variables included practice size, number of patient contacts, working hours, on-call duties, and involvement in other professional activities outside primary care practice. Other variables were sector (public/private), location (urban/rural), and solo or group (shared) practice.

### Statistical analysis

Data analysis included descriptive statistics, chi-square test, and logistic regression analysis. Multiple logistic regression analysis was used to determine factors that influenced the likelihood of job satisfaction outcomes. Alpha level of 0.05 was used for all statistical tests. A Bonferroni correction was applied for individual regression analysis of the six outcomes to avoid inflation of type 1 error, setting the significance level at 0.008 (*p* = 0.05/6). All analyses were performed using R 3.4.1 in RStudio (version 1.0.143) [37]. An exploratory analysis was conducted analysing the responses as continuous, ordinal, and binary variables. Since the results were similar, results of the binary outcome are shown for simplicity.

### Ethics

This study was approved by the Medical Research and Ethics Committee, Ministry of Health Malaysia as one of the components of the Malaysia Health System Research study (NMRR-15-607-25 769).

## Results

A total of 460 doctors participated in the study; 221 doctors were from public clinics and 239 doctors were from private clinics. Demographic characteristics of the participating doctors are shown in Table 1. Over half of the doctors in public clinics were female (61.5%) compared to only 34.3% in private. In public clinics, doctors aged under 30 years made up the largest age group, followed by those aged 31–40 years. In contrast, private doctors were older with around half of them (49.4%) aged over 50 years. While the number of public clinics was more or less evenly distributed between urban and rural areas, almost all of the private clinics (over 90%) surveyed were located in urban areas. Nearly half of the private doctors work alone as a solo practitioner in a clinic compared to only 16.3% of public doctors. On average, direct patient care accounted for 92.2% of the total working hours per week for doctors in public clinics and 95.4% of working hours for doctors in private clinics. Public doctors reported longer on-call hours in the last 3 months and a higher average frequency of visits to other primary care clinics than private doctors.

**Table 1:** Characteristics of primary care doctors

	Public (*n* = 221)	Private (*n* = 239)
Doctor characteristics		
Sex, *n* (%)		
Male	85 (38.5)	157 (65.7)
Female	136 (61.5)	82 (34.3)
Age group, *n* (%)		
≤ 30	139 (62.9)	3 (1.3)
31–40	72 (32.6)	42 (17.6)
41–50	9 (4.1)	76 (31.8)
51–60	1 (0.5)	59 (24.7)
> 60	–	59 (24.7)
Mean (SD)	30.7 (4.4)	51.2 (11.2)
Born in Malaysia, *n* (%)	213 (96.4)	226 (94.6)
Practice characteristics		
Practice size^a^, mean (SD)	60 546.7 (55 959.0)	15 153.3 (9 652.0)
Location, *n* (%)		
Urban	101 (45.7)	224 (93.7)
Rural	120 (54.3)	15 (6.3)
Solo practice, *n* (%)	36 (16.3)	108 (45.2)
Working hours per week, mean (SD)	41.0 (2.0)	37.2 (7.4)
Hours spent per week on direct patient care, mean (SD)	37.8 (4.9)	35.5 (8.1)
Number of patient contact per day, mean (SD)		
Face-to-face	43.1 (21.4)	39.6 (18.8)
By telephone	1.2 (2.5)	1.6 (2.3)
Number of on-call duties in the past 3 months, mean (SD)		
Evening (1700–2200 hours)	5.1 (7.0)	11.0 (9.1)
Overnight (2200–0800 hours)	4.4 (7.3)	1.0 (3.9)
Weekend	2.0 (2.7)	3.7 (2.9)
Total	11.5 (16.3)	15.7 (11.5)
Total on-call hours in the past 3 months, mean (SD)	106.0 (163.6)	63.0 (53.6)
Number of out-of-office visits per week, mean (SD)		
Other primary care clinic	17.5 (37.1)	6.4 (25.2)
Home visit	0.1 (1.1)	0.4 (1.2)
Institutions (school, orphanage, community centre)	3.4 (16.0)	1.0 (6.0)
Average consultation time (minutes), mean (SD)	11.2 (5.2)	12.6 (5.4)

*Abbreviation*: *SD* standard deviation

^a^Estimated practice size is based on the total number of patients visited the clinics in the previous year

The overall job satisfaction score was calculated by taking the average (mean) scores of the six items as defined in the “Methods” section. The overall mean score was 2.95 (SD 0.42); the mean score from public doctors was 2.81 (SD 0.41) while the mean score from private doctors was slightly higher at 3.07 (SD 0.39). Table 2 shows a comparison of responses for the job satisfaction variables between public and private doctors. Overall, the administrative task appears to be the aspect doctors in public and private clinics have the most problems with. The largest absolute difference between public and private doctor responses was observed for the variables concerning administrative task and effort-reward balance. Well over half of the doctors in public clinics (59.7%) agree that the work was overloaded with unnecessary administrative task as opposed to only 36.0% of the doctors in private clinics (*p* < 0.001). In terms of the balance between effort and reward, 62.9% of public doctors agree that there was a good balance while a significantly higher proportion (85.8%) of private doctors provided the same response (*p* < 0.001). Approximately one third of the doctors in public clinics agree that parts of their work do not make sense and the proportion was significantly lower among private doctors (18.4%). On the question “I have too much stress in my current job”, just less than a quarter of doctors in both public and private clinics agree with the statement; however, the differences between sectors were only significant among doctors whose clinic is in urban areas. A great majority (over 80%) of doctors in both public and private clinics agreed that being a doctor is a well-respected job. Moreover, over 90% agree that their job still interests them as much as it ever did.

**Table 2:** Job satisfaction of primary care doctors

Variable (% responding “agree” or “strongly agree”)	Public (*n* = 221)	Private (*n* = 239)	*p* value	Mean Difference (95% CI)
I feel that some part of my work do not really make sense	33.9	18.4	**< 0.001**	0.16 (0.08, 0.23)
Urban	41.2	19.2	**< 0.001**	0.22 (0.11, 0.33)
Rural	27.7	6.7	0.08	0.21 (0.06, 0.36)
My work still interest me as much as it ever did	93.7	97.5	**0.04**	−0.04 (− 0.08, < 0.01)
Urban	92.2	97.3	**0.03**	−0.05 (− 0.10, < 0.01)
Rural	95.0	100.0	0.38	−0.05 (− 0.16, 0.06)
My work is overloaded with unnecessary administrative detail	59.7	36.0	**< 0.001**	0.24 (0.15, 0.33)
Urban	66.7	36.6	**< 0.001**	0.30 (0.19, 0.41)
Rural	53.8	26.7	**0.05**	0.27 (0.03, 0.51)
I have too much stress in my current job	23.1	17.6	0.14	0.06 (−0.02, 0.13)
Urban	36.3	17.9	**< 0.001**	0.18 (0.08, 0.29)
Rural	11.8	13.3	0.86	−0.02 (− 0.20, 0.17)
Being a doctor is a well-respected job	84.6	87.9	0.31	−0.03 (− 0.10, 0.03)
Urban	79.4	87.5	0.06	−0.08 (− 0.17, 0.01)
Rural	89.1	93.3	0.61	−0.04 (− 0.18, 0.10)
In my work there is a good balance between effort and reward	62.9	85.8	**< 0.001**	−0.23 (− 0.31, − 0.15)
Urban	52.9	85.3	**< 0.001**	−0.32 (− 0.43, − 0.22)
Rural	71.4	93.3	0.07	−0.22 (− 0.37, − 0.07)

Bold font denotes significance at *p* < 0.05

*Abbreviation*: *CI* confidence interval

**Fig. 1:**
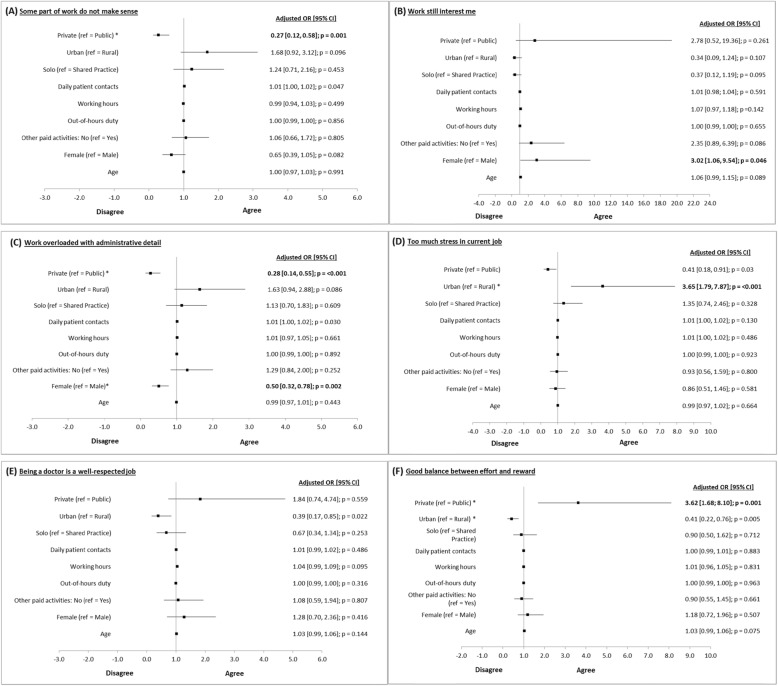
Logistic regression analysis of factors associated with the job satisfaction variables analysed separately for the six items: **a** Some part of work do not make sense, **b** Work still interest me, **c** Work overloaded with administrative detail, **d** Too much stress in current job, **e** Being a doctor is a well-respected job, **f** Good balance between effort and reward. Abbreviations: CI = confidence interval, OR = odds ratio

Figure 1 shows a summary of six multiple logistic regressions examining the predictive utility of the sociodemographic, practice, and workload factors separately for each of the work satisfaction variables (Fig. 1). After adjustment for other factors, namely age, gender, practice location, type of practice, patient load, working hours, on-call duties, and involvement in other professional activities outside primary care practice, differences between sectors remained significant for three of the work satisfaction variables: “parts of work do not make sense”, “work overloaded with administrative task”, and “balance between effort and reward”. Practice location in an urban area was significantly associated with outcomes on “stress in the current job” (OR 3.7; CI 1.8 to 7.9) and “good balance between effort and reward” (OR 0.4; CI 0.2 to 0.8). For all six items of the work satisfaction variables, women tended to report more positively than men but the differences did not reach statistical significance in all instances except for the burden of administrative task. Furthermore, work characteristic variables, i.e. patient load, working hours, on-call duties, and solo practice, did not show a significant association with all aspects of work satisfaction (Fig. 1).

We conducted a sensitivity analysis by including only doctors working in urban areas in the multiple regression model. We observed similar findings where differences between public and private sectors persisted for the variables “part of work do not make sense”, “job too stressful”, and “good balance between effort and reward”.

## Discussion

This study measures the perception of job satisfaction among doctors of the public and private primary care clinics in Malaysia and identifies factors significantly associated with the outcomes of interest.

Overall, our results show that primary care doctors in Malaysia appear to be quite satisfied with their work. Comparison of our findings to an earlier study by Stobbe [38] on GP’s job satisfaction in 34 countries from the QUALICOPC study reveals that the mean scores derived from the Malaysian doctors are relatively high (Table 3). This is an interesting finding since countries with high GP job satisfaction scores are the wealthier western countries with a relatively stronger primary care system and health workforce density of about three to four times than Malaysia [39]. Our study shows that primary care doctors in Malaysia have had an average of 40 consultations per day, less than 15 min of consultation time, and practice size of over 15 000. This is well above the recommendation by a recent study which indicated that in many European countries, general practice workload is considered reasonable and sustainable when they have less than 25 face-to-face consultations a day, spend more than 20 min for consultation, and have a practice list size of 1600 or fewer [40]. Yet, the mean job satisfaction scores of doctors in Malaysia are higher than most of these European countries. It can be assumed that the expectations of primary care doctors differ from country to country due to differences in demand, tasks, and activities. However, there is also reason to believe that fundamental cultural differences may be at play. Two recent multi-country studies (24 and 48 countries) showed that job satisfaction was significantly moderated by national culture [41, 42]. In the field of primary care, the doctor-patient relationship determines job characteristics, and these are subject to cultural artefacts such as “power distance” and views on “individualism/collectivism”. In one 10-country study of 307 primary care doctors, in those countries where doctors were in a more culturally authoritative position, consultations tend to be shorter and the information exchange better-matched patient expectations [43]. Meeting patient expectations in primary care consultations appears to be associated with greater job satisfaction [44], and the greater authority of a Malaysian doctor may, speculatively, help them achieve this. Without further research, it would be hard to draw a definitive conclusion, but it may help to explain what on its surface appears to be an anomalous result.

**Table 3:** Comparison of mean job satisfaction scores between countries

Country	Mean job satisfaction score	Country	Mean job satisfaction score
Denmark	2.97	England	2.49
Malaysia^a^	2.95	Malta	2.47
Cyprus	2.81	Germany	2.45
Canada	2.77	Bulgaria	2.44
Norway	2.75	Portugal	2.41
Sweden	2.73	Poland	2.41
Australia	2.72	Romania	2.38
Luxembourg	2.71	Italy	2.37
Switzerland	2.69	Latvia	2.36
New Zealand	2.68	Macedonia	2.35
Netherlands	2.63	Turkey	2.30
Greece	2.62	Slovenia	2.29
Ireland	2.60	Lithuania	2.27
Belgium	2.59	Estonia	2.27
Finland	2.59	Slovakia	2.23
Austria	2.56	Hungary	2.17
Iceland	2.50	Spain	2.15
Czech Republic	2.49		

Reproduced with permission from “Job satisfaction of general practitioners: an international comparison” by E. Stobbe, 2018 [27]

^a^Data from this study

The results suggest that doctors in public primary care clinics had lower job satisfaction than their counterparts in private clinics. This could be attributed to the different governance in the two sectors in Malaysia. Private clinics consist of small practices with a single practitioner or few with group practice (Table 1). As such, doctors can have more control and flexibility in the daily running of clinics and work freedom [13, 45]. Considering that public healthcare is funded and centrally managed by the government, doctors in public clinics are saddled with more responsibility but at no extra remuneration. On top of attending to patients, they are often delegated many additional functions such as preparing paperwork, performance measures and reporting, documentation requirements, or attending meetings [5, 23]. Although these tasks may seem minor, doctors could perceive it as overwhelming and burdensome which resulted in unfavourable responses. This is reflected in the current study where public doctors were more likely to feel dissatisfied with clerical work and irrelevant tasks assigned. Other possible explanation for the differences in the satisfaction level between public and private doctors could be due to the preponderance of younger doctors in public clinics. Several studies show a relationship between age and job satisfaction where older age is associated with greater job satisfaction among doctors [21, 46]. Older doctors are usually more experienced and thus tend to be more comfortable or used to current work conditions which may lead to a greater satisfaction rate. Nevertheless, the effect of age on job satisfaction among the primary care doctors was not significant in the present study.

In terms of individual job satisfaction measures, our study identifies a substantial proportion of primary care doctors who felt some part of their work do not make sense and considered that their work is being overloaded with unnecessary administrative task. Our results echoed past studies where doctors have numerously expressed dissatisfaction with administrative responsibilities in their daily job [27, 28, 47]. Studies among doctors in the United States show that time spent on administrative tasks ranged between 16 and 24% of their total work hours [48, 49]. This highlights the fact that primary care doctors’ daily work routine consists of various tasks that may fall outside of their professional role which could be regarded as unreasonable or unnecessary. Besides, Thun et al. have shown that administrative task to be closely associated with unreasonable task load as perceived by the doctors [50]. The issue with understanding this area is that there are many different intents that drive these tasks; for instance, it could be requirements by the clinic itself such as patient documentation and test results or from outside clinics such as performance measures and insurance-related matters. Generally, doctors view the need to complete the desk job as worthwhile if it adds high value to patient outcome [51]. As such, the effect of these documentations to the overall patient care and institutional growth will have to be explored further.

Comparing our results regarding the work satisfaction measures with the study published by Hoffman et al. and Butu et al. from the similar framework of the QUALICOPC study, a higher level of dissatisfaction with administrative workload was reported among general practitioners in Austria and Romania [36, 47]. Another predominant response from Austria’s and Romania’s general practitioners was on the high level of stress; yet, only less than one quarter of Malaysian primary care doctors reported the same. These differences could be related to different primary care practice setting and work content between countries, though it may also reflect the greater acceptance by Malaysian doctors to non-clinical task load and ability to tolerate job-related stress. Nevertheless, our results show that most of the primary care doctors from both sectors do find value in their job as majority expressed continued interest in their job, expressed satisfaction with effort-reward balance, and regarded the profession as a well-respected job.

### Strengths and limitations

To the best of our knowledge, this is the first study assessing the job satisfaction of primary care doctors in both the public and the private sector in Malaysia. Minimum sample size requirement for the QUALICOPC was met for both public and private sectors; therefore, we are able to make a direct comparison on levels of job satisfaction between the public and private sector. It also benefited from the use of a standard, well-developed QUALICOPC questionnaires used in many other countries whereby we can benchmark our results with other countries that have participated in the QUALICOPC study. Nevertheless, this study has some limitations. It was a cross-sectional study, and it is not possible to infer causal links of the findings. This study examined job satisfaction of the primary care doctors as part of a larger QUALICOPC study, and the six questions may not be a comprehensive tool to measure job satisfaction in finer details. However, it can be considered a cost-effective approach in which job satisfaction component is integrated within the QUALICOPC questionnaire and researcher can also capture this information when rolling out the QUALICOPC study.

## Conclusion

In a time when health reform has put a spotlight on primary health care service delivery and workforce, job satisfaction of the primary care doctors is an important component to address as part of the puzzle towards enhancing the quality of the healthcare. In all, this study showed that primary care doctors in Malaysia were moderately satisfied with their job. Sector differences in job satisfaction did exist among primary care doctors. A higher proportion of public doctors experienced pressure from administrative tasks and felt that part of their work does not make sense than their colleague in the private sector. At the same time, the majority of private doctors reported positive outcome on effort-and-reward balance compared to only one third of public doctors. Result of this study also showed that doctors practising in urban areas were more likely to experience stress in the current job. This study suggests that decreasing administrative workload and increasing work-based supports might be the most effective ways to improve the job satisfaction of primary care doctors because these are some of the main aspects of the job that doctors, especially in public clinics, are most unhappy with. Although non-clinical-related activities such as administrative task are part of the doctors’ responsibilities, the balance between these activities and direct patient care may need to be addressed.

### Policy implications

The findings of this study present several opportunities for policymakers and healthcare institutions to work towards addressing the needs of primary care doctors in the country. Enhancement of job satisfaction at the primary care level can build up employee motivation and efficiency, which may encourage them to stay and improve the retention rate of public primary care physician. By improving workplace systems, processes, and environment, it could increase the attractiveness of working in the public sector and curb the migration from the public to private sector. Currently, at least in rhetoric, there is a government initiative for public-private partnership (PPP) in the health sector to address service delivery and workforce challenges [4, 52]. Hence, the findings obtained from this study will be useful for identification of enabling factors for successful and sustainable implementation of PPP in the country as we progress towards meeting the health system goals and sustaining UHC.

## Supplementary information

**Additional file 1.** QUALICO-PC questionnaire for doctors.

## Data Availability

All data supporting the findings are included in this manuscript. Additional data are available from the corresponding author on reasonable request.
