# Supplementary material for: Job satisfaction of public and private primary care physicians in Malaysia: analysis of findings from QUALICO-PC
Source: Hum Resour Health. 2019 Nov 4;17:82. doi: 10.1186/s12960-019-0410-4 (PMC6829856; doi:10.1186/s12960-019-0410-4)
Supplement: Supplementary file 1 — Additional file 1. QUALICO-PC questionnaire for doctors. [file 12960_2019_410_MOESM1_ESM.pdf]

QUALITY AND COSTS OF PRIMARY CARE (QUALICO-PC)

Questionnaire for Doctors

Name of Clinic:	
Interview done by:	
Time start:	

No	Question	Response categories	Remark
1	Are you male or female?	O Male	
		O Female	
2	What is your year of birth?<br>Please fill in:	Year of birth: 19____	
3	Were you born in this country?	O Yes	
		O No	
4	How would you characterise the place where you are currently practising?	O Big city	Changed from big (inner) city
		O Suburbs	
		O (Small) town	
		O Mixed-urban rural	
		O Rural	
5	To what extent do you think your practice population compares to the average national level with respect to the following categories:		
	1. Elderly people (over 70 years)<br>*[NMCS 2014: 5.7%]*	O Below average<br>O Average<br>O Above average<br>O Don't know	
	2.Socially disadvantaged people<br>*[Eligible for BR1M or monthly income per household < RM4000]*	O Below average<br>O Average<br>O Above average<br>O Don't know	
	3. Ethnic minority people<br>*[Other than Malay/Chinese/Indian/Iban/Kadazan]*	O Below average<br>O Average<br>O Above average<br>O Don't know	

	4. Elderly people (over 60 years)<br>*[NMCS 2014: 15%]*	O Below average<br>O Average<br>O Above average<br>O Don't know	
	5. Foreigners<br>*[NMCS 2014: 6.2%]*	O Below average<br>O Average<br>O Above average<br>O Don't know	
6	To what extent do you think that the patient turnover in your practice compares to other practices in this country?	O Below average<br>O Average<br>O Above average<br>O Don't know	
7	How many hours per week do you work as a medical doctor (<u>excluding</u> additional jobs and on- call or out-of-hours services)?	____ hours per week	Between 8am-5pm
8	How many of these hours do you spend on direct patient care (consultations, home visits, telephone consultations)?	____ hours per week	Maximum is the number filled in question 7
9	How many patient contacts do you have on a normal working <u>day?</u>		
	1. Face-to-face in your office (number)	____ per day	
	2. By telephone	____ per day	
	3. By email	____ per day	
10	How long does a regular patient consultation in your office usually take?	____ minutes	To give an estimate of average duration
11	In a normal working <u>week,</u> how many patients do you see:		
	1. At home visits	____ per <u>week</u>	
	2. In hospital	____ per <u>week</u>	
	3. In homes for the elderly	____ per <u>week</u>	
	4. In other institutions or settings	____ per <u>week</u>	Include visits to school, orphanages, pemulihan komuniti , domiciliary care, centres for disabled
	5.In other clinics	____ per <u>week</u>	Locum and extended hours

12	In the past working month (excluding holidays etc.), how often and for how long did you have on-call duties during evenings, nights and weekends:		
	1. During evening(s)	____ times: in total ___hours	5pm to 10pm
	2. During night(s)	____ times: in total ___hours	10pm until next morning
	3. During weekend days	____ times: in total ___hours	
13	Beside your work as a medical doctor in this practice, do you have any other paid professional activities? (multiple answers possible)	☐ No	
		☐ Yes, as a physician for privately paying patients	For doctors who provide personal care to specific patients
		☐ Yes, in a residential setting (e.g. nursing home, prison)	
		☐ Yes, as a company doctor	Company doctor- doctor who makes visit to the workplace
		☐ Yes, in teaching/medical education	
		☐ Yes, for extended hours in the public clinics	
		☐ Yes, for out-of hours service in private clinics	Locum in private clinics
		☐ Yes, other<br>Please specify:____________	
14	As a medical doctor, are you self-employed or in salaried employment? (multiple answers possible)	☐ Salaried employment with centre or authority	In KK, click this answer
		☐ Salaried employment with other medical doctors	For doctors who work in a private clinic and getting salary from the clinic
		☐ Self-employed with contract(s) with health service, insurance or authority	For panel doctor who owns the clinic, click this answer
		☐ Self-employed without contract	For GPs who depend totally on walk-in patients

15	For each of the following components please estimate whether they contribute to your income as a medical doctor, and if so, up to what percentage? (multiple answers possible)	☐ Salary ____%			
		☐ Capitation payments (a fixed sum per patient for a certain period of time) ____%			Payment given to perform a fixed list of procedures
		☐Fee for services from third party payer ____%			For Managed Care Organisation(MCO) such as SOCSO or Insurance
		☐Out-of-pocket payments from patients ____%			
		☐ Performance payments (for instance related to targets) ____%			
		☐ On-call claim from extended hours in the public clinics_____%			For those providing services in the public clinics after office hours
		☐ Out-of hours service in private clinics______%			For those providing services in the private clinics after office hours
		☐Other sources ____			
16	Can you receive an extra financial incentive or bonus for:	**Yes**	**No**	**Don't Know**	
	1. Management of patients with diabetes	○	○	○	
	2. Management of patients with hypertension	○	○	○	
	3. Achievement of targets for screening or prevention	○	○	○	
	4. Referral rates below a certain level	○	○	○	
	5. Having disadvantaged patients in your practice	○	○	○	
	6. Working in a remote area	○	○	○	

17	Do you work alone or in shared accommodation with one or more medical doctors and/or medical specialists?<br>If part time, please also fill in the total number of hours.<br>(multiple answers allowed)<br>*Include all doctors working in this clinic	☐Alone		
		☐With other medical doctors/family medicine specialist in shared accommodation	How many in total (including self):____<br><br>How many full time:<br>_____<br><br>For part time:<br>____total working hours (in a week)	
		☐With medical specialist(s) in shared accommodation	How many in total (including self if 2nd option not ticked):<br>____<br><br>How many full time:<br>____<br><br>For part time:<br>____total working hours (in a week)	Not to include FTE of the surveyed doctor if above is ticked<br>Medical specialist- any specialist except Family Medicine Specialist
18	Which of the following disciplines are working in your practice/centre?<br>(Multiple answers allowed)	**Yes**		To include visiting personnel
	1. Receptionist/medical secretary	☐		Include clerks
	2. Practice Nurse / Assistant Medical Officers	☐		Staff nurses with diploma/degree in nursing/AMO
	3. Community/home care nurse	☐		Jururawat masyarakat in public sector

	4. Psychiatric nurse:<br>4.1 with post-basic<br>4.2 informal training	☐<br>☐	
	5. Nurse practitioner<br>(function between physician and nurse)	☐	A nurse practitioner is a nurse with a Master's degree. He/she functions between a doctor and practice nurse who provides a complete episode of care to patients of any age (This includes patient examination and making clinical decisions on treatment)
	6. Assistant for laboratory work	☐	Include Medical Lab Assistant (MLT)
	7. Manager of the centre or practice (not a physician)	☐	Administrator
	8. Midwife	☐	
	9. Physiotherapist	☐	
	10. Dentist	☐	
	11. Pharmacist	☐	
	12. Social worker	☐	
	13. Nursing aid	☐	Working as nurse with little or without formal training. Qualification for nursing aides: SPM, STPM, Diploma/Degree (Any program unrelated to nursing program)
	14. TB nurse	☐	Nurses with **formal training (post basic training)** in the respective field, helping doctors to educate patients and ensure patient's adherence to the management prescribed

	15. Chronic disease nurse	☐	Nurses with **formal training (post basic training)** in the respective field, helping doctors to educate patients and ensure patient's adherence to the management prescribed
	16. HIV counsellor	☐	Nurses with **formal training (post basic training)** in the respective field, helping doctors to educate patients and ensure patient's adherence to the management prescribed
	17. Methadone maintenance nurse	☐	Nurses with **formal training (post basic training)** in the respective field, helping doctors to educate patients and ensure patient's adherence to the management prescribed
	18. Occupational therapist	☐	
	19. Dietician	☐	A dietitian is a health professional who has university qualifications consisting of a 4-year Bachelor Degree in Nutrition and Dietetics or a 3-year Science Degree followed by a Master Degree in Nutrition and Dietetics, including a certain period of practical

					training in different hospital and community settings. They may work with people who have special dietary needs, inform the general public about nutrition, evaluate and improve treatments and educate clients, doctors, nurses, health professionals and community groups.
	20. Nutritionist	☐			A nutritionist is a non-accredited title that may apply to somebody who has done a short course in nutrition. Nutritionists do not have any professional practical training, and therefore they should not be involved in the diagnosis and dietary treatment of any diseases.
	21. Medication Dispenser	☐			
	22. Radiographer	☐			
19	Do you use clinical guidelines for the treatment of the following?	**Yes**	**No**	**Guidelines Not Available**	
	1. Chronic heart failure	○	○	○	
	2. Asthma	○	○	○	
	3. COPD	○	○	○	COPD- chronic obstructive pulmonary disease
	4. Diabetes	○	○	○	

20	In the past 12 months, have you been involved in a disease management programme for patient with the following chronic conditions? (Such programmes are multidisciplinary approaches across practices, often based on protocols).	**Yes**	**No**	A disease management program will consist of registry, appointment system and has to be a multidisciplinary care(the multidisciplinary care may be provided outside this clinic) Eg: Antenatal/postnatal care in MCH clinics or TB cases involving other medical disciplines
	1. Chronic heart failure	O	O	
	2. Asthma	O	O	
	3. COPD	O	O	
	4. Diabetes	O	O	
	5.Human Immunodeficiency Virus (HIV)	O	O	
	6.Methadone Maintenance Therapy	O	O	
	7. Maternal and Child Health	O	O	
	8. Psychosocial Rehabilitation	O	O	
21	In the past 12 months, has the following occurred in your practice/centre?	**Yes**	**No**	
	1. Feedback on your prescriptions or referrals by health authority or insurer?	O	O	Include state level feedback on prescription intervened and prescription error
	2. Feedback from colleague medical doctors (peer review or practice visitation)?	O	O	Include visiting FMS

	3. Investigation into the satisfaction of your patients?	O	O	Include Klinik Kawanku patient satisfaction survey in public sector
	4. Feedback from in-house pharmacists	O	O	

22	In case of referral, who usually decides about where the patient is referred to?	O I do	
		O The patient does	
		O It is a shared decision	

23	In case of referral, to what extent do you take into account the following considerations:	**Always**	**Sometimes**	**Never**	
	1. The patient's preference where to go	O	O	O	
	2. The travel distance for the patient	O	O	O	
	3. Your previous experiences with the medical specialist	O	O	O	
	4. Comparative performance information on medical specialists.	O	O	O	
	5. Waiting time for the patient	O	O	O	
	6. Costs for the patient	O	O	O	

24	Please tick the equipment used in your practice by yourself or your staff (Multiple answers allowed):		
	**Laboratory**	**Functions**	
	O Haemoglobinometer<br>-Instrument used to determine the haemoglobin level of blood	O Audiometer<br>-A machine used for evaluating hearing loss	
	O Any blood glucose test set	O Bicycle ergometer<br>-A stationary bicycle to measure the work done by the exercises	
	O Any cholesterol meter	O Eye tonometer<br>-Instrument used to measure the pressure inside the eye	
	O Blood cell counter<br>- full blood count point of care test	O Peak flow/PEF meter	

	○ Bilirubinometer	○ Spirometer	
	○ HbA1c analyser	○ Electrocardiograph	
	○ Haematological analyser<br>-Instrument used to perform complete (full) blood count	○ Blood pressure meter (BP set)	
	○ Biochemistry analyser<br>-Instrument used to determine concentration of metabolites and electrolytes	○ Infusion set	
		○ Doctor's bag for emergencies and home visits	
	**Imaging**	**Other**	
	○ Ophthalmoscope	○ Urine catheter	
	○ Proctoscope	○ Coagulometer (cautery)	
	○ Otoscope	○ Set for minor surgery	
	○ Gastroscope	○ Suture set	
	○ Sigmoidoscope	○ Defibrillator	
	○ X-ray	○ Disposable syringes	
	○ Ultrasound for abdomen/foetus	○ Disposable gloves	
	○ Microscope	○ Refrigerator for medicines	
		○ Resuscitation equipment	
		○ Nebuliser	
		○ Alternative birthing Centre (ABC)	
		○ Dapton	A hand held, portable ultrasound tool used for fetal heart monitoring
		○ Fundus camera	
25	How do you have access to laboratory facilities?	○ Within my practice/centre<br>○ Easy access outside my practice/centre<br>○ Insufficient access	A laboratory service within clinic facility with dedicated personnel
		○ Within my practice/centre	

26	How do you have access to X-ray facilities?	O Easy access outside my practice/centre	
		O Insufficient access	
27	What is the distance by road from your(main) practice building to:		
	1. The nearest clinic doctor practice (not in your group or centre)	O In the same building<br>O Less than 10 km<br>O 11-20 km<br>O More than 20 km	Includes Klinik Kesihatan with doctors (medical officer/family medicine specialist): Private clinics And Family Medicine Specialist.
	2. The nearest consultant/outpatient clinic (independent or part of hospital)	O In the same building<br>O Less than 10 km<br>O 11-20 km<br>O More than 20 km	Specialist clinics except Family Medicine Specialist clinics
	3. The nearest general or university hospital	O In the same building<br>O Less than 10 km<br>O 11-20 km<br>O More than 20 km	
28	How many hours on an average working day is your practice/centre open for patient care<br>(lunch breaks excluded)?	____ hours per working day	Also includes any breaks observed by the practice during working hours.
29	Is it possible for your patients to visit your practice/centre:		
	1. After 18.00h (at least once per	O Yes	

	week)	O No	
	2. On a weekend day (at least once per month)	O Yes	
		O No	
30	During evenings and nights at <u>weekdays</u>, how do your patients have access to (non- emergency) medical services?	O I am always available for my patients (Not applicable)	
		O I am available on a rota basis with a group of primary care doctors	This doctor rotates with others to provide out-of-hours care within the same geographical area
		O I am not available, but other primary care doctors are available (on a rota basis)	There are doctors rotating among themselves to provide out-of-hours care within the same geographical area **but this doctor does not participate in it**
		O Other physicians (not primary care doctors) provide out-of-hours care	The patients in the geographical area visit other doctors besides primary care doctors (e.g. hospital based doctors)
		O Other arrangements	
31	On weekends, how do your patients have access to (non- emergency) medical services?	O I am always available for my patients (Not applicable)	
		O I am available on a rota basis with a group of primary care doctors	This doctor rotates with others to provide out-of-hours care within the same

			geographical area
		O I am not available, but other primary care doctors are available (on a rota basis)	There are doctors rotating among themselves to provide out-of hours care within the same geographical area but this doctor does not participate in it
		O Other physicians (not primary care doctors) provide out-of-hours care	The patients in the geographical area visit other doctors besides primary care
		O Other arrangements	
32	What percentage of your patient consultations is by appointment?	About ____ %	
33	Do you offer a walk-in hour?	O Yes	
		O No	
34	In the past 12 months, have you ever done the following to reduce financial obstacles to disadvantaged patients:		
	1. Provide free samples of medication	O Yes	Not applicable for KK
		O No	
	2. Prescribe the cheapest equivalent medicine	O Yes	Not applicable for KK
		O No	
	3. Not charge the patient (e.g. for co-payments)	O Yes	
		O No	
	4. Refer to social welfare	O Yes	
		O No	
35	In the past 12 months, how	O Frequently	Includes

	often have you noticed that patients delayed their visits for financial reasons?		patients who cannot travel to clinic due to financial reasons
		○ Occasionally	
		○ Never	
36	If new patients enter your practice, do you receive their medical records from their previous doctor?	○ Yes, always or usually	Medical records to include discharge summaries , Teleprimary care records and referral letters
		○ Only occasionally	
		○ Rarely or never	
37	Which restrictions do you apply to accepting new patients? (More than one answer possible)	☐ No restrictions (everyone is accepted)	Public Sector only allowed to answer the first answer
		☐ No new patients are taken above a maximum number	
		☐ No new patients are taken above a certain age	
		☐ No new patients are taken outside my geographical working area	
		☐ I use a wait period for new patients	
		☐ Acceptance depends on patients' medical history	
		☐ Acceptance depends on patients' insurance status	
38	Do you provide health care to people, when you are not remunerated for this (for instance uninsured, illegal immigrants)?	○ Yes, (almost) always	
		○ Yes, but only in urgent cases	
		○ Yes, sometimes	
		○ No	
		○ No such people show up in my practice	
		○ Not applicable (in this country such care is remunerated)	
		○ Not applicable (costs are covered by the government)	For public clinics, please tick this

39	Do your medical files normally include the following information: (Tick all that apply)	☐Living situation	Includes social history: E.g Stay alone/with families, lodging condition and sanitation
		☐Ethnicity	
		☐Patients' family history (e.g. depression, cancer)	
		☐Patients' weight and height	
		☐Smoking	
		☐Blood pressure	
		☐Reason for encounter	Reason for visit/chief complaint
		☐Diagnosis	
		☐Prescribed medications	
		☐Test results	
40	How do you keep patient medical records? (Tick all that apply)	☐I keep records except for minor or trivial complaints	
		☐I only keep records of regularly attending patients	
		☐I keep records , unless it is too busy	
		☐I keep records routinely of all patient contacts	For public clinics, please tick this
		☐Don't know	

41	In the past 2 years, have you used your medical record system to list a selection of patient on the basis of age, diagnosis or risk? (Tick all that apply)<br>Both manual and electronic	☐No	Both manual and electronic
		☐Yes, by age (e.g. those above age 50)	
		☐Yes, by diagnosis or health risk (e.g. diabetes or hypertension)	For public clinics, check if clinics are involved in registries. If yes, tick this
		☐Yes, by medications they take (e.g. patients on multiple medications)	
		☐Yes, to send reminders for prevention or follow-up	
		☐I don’t know	

42	For which of the following purposes do you use a computer in your practice<br>(Tick all that apply)	☐Not applicable (I don't use a computer)	
		☐Making appointments	
		☐Issuing invoices	
		☐Issuing drug prescriptions	
		☐Keeping records of consultations	
		☐Sending referral letters to medical specialists	
		☐Storing diagnostic test results	
		☐Searching medical information on the internet	
		☐Sending prescriptions to the pharmacy	

43	How often do you meet face-to-face with the following professionals (either professionally or socially):	**Seldom or never**	**Every 1-3 months**	**More than once a month**	
	1. Other medical doctors	o	o	o	Exclude medical specialist
	2. Practice nurse	o	o	o	Staff nurses with diploma/degree in nursing
	3. Ambulatory medical specialist	o	o	o	Other specialist from a clinic **besides** family medicine specialist
	4. Hospital medical specialist	o	o	o	
	5. Pharmacist	o	o	o	
	6. Home care nurse	o	o	o	
	7. Midwife	o	o	o	
	8. Physiotherapist	o	o	o	
	9. Social worker	o	o	o	
	10. Dietician	o	o	o	
	11. Family medicine specialist	o	o	o	

44	How often do you ask advice (e.g. by telephone) from the following medical specialists?	**Seldom or never**	**Every 1-3 months**	**More than once a month**	
	1. Paediatrician	o	o	o	

	2. Internist or physician (one with MRCP qualification)	O	O	O	
	3. Gynaecologist	O	O	O	
	4. Surgeon	O	O	O	
	5. Neurologist	O	O	O	
	6. Dermatologist	O	O	O	
	7. Geriatrician	O	O	O	
	8. Psychiatrist/ mental health professional	O	O	O	
	9. Radiologist	O	O	O	
45	Does your practice nurse (staff nurse and assistant medical officer) or assistant independently provide:	O Not applicable (No nurse in my practice)			
	1. Immunisation	O Yes			
		O No			
	2. Health promotion (e.g. giving lifestyle or smoking cessation advice)	O Yes			
		O No			
	3. Routine checks of chronically ill patients (e.g. diabetes)	O Yes			
		O No			
	4. Minor procedures (e.g. ear syringing, wound treatment)	O Yes			
		O No			
46	To what extent do you use referral letters **(including details on provisional diagnosis and possible test results)** when you refer patients to a medical specialist? I use letters:	O For all patients that I refer			
		O For most patients that I refer			
		O For a minority of patients that I refer			
		O Seldom or never			
47	To what extent do medical specialists inform you after they have finished the treatment or diagnostics of your patients?	O (Almost) always			
		O Usually			
		O Occasionally			
		O Seldom or never			

48	After a patient has been discharged, how long does it usually take to receive a (summary) discharge report from the hospital most frequented by your patients?	O 1-4 days				This question is asking if you receive discharge report directly from the hospital. If yes, how long does it take to get the discharge report.
		O 5-14 days				
		O 15-30 days				
		O More than 30 days				
		O I rarely or never receive a discharge report				
49	In case of the following health problems, to what extent will patients in your practice population (people who normally apply to you for primary medical care) contact you as the first health care provider?<br>(This is only about the **first contact**, not about further diagnosis or treatment).	**(Almost) Always**	**Usually**	**Occasionally**	**Seldom or Never**	
	1.Child with severe cough	O	O	O	O	
	2. Child aged 8 with hearing problem	O	O	O	O	
	3. Woman aged 18 asking for oral contraception	O	O	O	O	
	4. Man aged 24 with stomach pain	O	O	O	O	
	5. Man aged 45 with chest pain	O	O	O	O	

	6. Woman aged 50 with a lump in her breast	o	o	o	o	
	7. Woman aged 60 with deteriorating vision	o	o	o	o	
	8. Woman aged 60 with polyuria	o	o	o	o	
	9. Woman aged 60 with acute symptoms of paralysis/paresis	o	o	o	o	
	10. Man aged 70 with joint pain	o	o	o	o	
	11. Woman aged 75 with moderate memory problems	o	o	o	o	
	12. Man aged 35 with sprained ankle	o	o	o	o	
	13. Man aged 28 with a first convulsion	o	o	o	o	
	14. Anxious man aged 45	o	o	o	o	
	15. Physically abused child aged 13	o	o	o	o	
	16. Couple with relationship problems	o	o	o	o	
	17. Woman aged 50 with psychosocial problems	o	o	o	o	
	18. Man aged 32 with sexual problems	o	o	o	o	
	19. Man aged 52 with alcohol addiction problems	o	o	o	o	
50	To what extent are you involved in the treatment and follow-up of patients in your practice population with the following diagnoses ('practice population' means: people who normally apply to you for primary medical care)?	**(Almost) Always**	**Usually**	**Occasionally**	**Seldom or Never**	
	1. Chronic bronchitis/ COPD	o	o	o	o	
	2. Hordeolum (Stye)	o	o	o	o	
	3. Peptic ulcer	o	o	o	o	
	4. Herniated disc lesion	o	o	o	o	
	5. Congestive heart failure	o	o	o	o	
	6. Pneumonia	o	o	o	o	
	7. Peritonsilar abscess	o	o	o	o	
	8. Parkinson's disease	o	o	o	o	
	9. Uncomplicated diabetes (type II)	o	o	o	o	

	10. Rheumatoid arthritis	o	o	o	o	
	11. Depression	o	o	o	o	
	12. Myocardial infarction	o	o	o	o	
51	To what extent are the following activities carried out in your practice population by you (or your staff) and not by a medical specialist? (Practice population means: people normally applying to you for primary medical care). For example, if fundoscopy is (almost) always done by you, tick that box	**(Almost) Always**	**Usually**	**Occasionally**	**Seldom or Never**	
	1. Wedge resection of ingrown toenail	o	o	o	o	
	2. Removal of sebaceous cyst from the hairy scalp	o	o	o	o	
	3. Wound suturing	o	o	o	o	
	4. Excision of warts	o	o	o	o	
	5. Insertion of IUD	o	o	o	o	
	6. Fundoscopy	o	o	o	o	
	7. Joint injection	o	o	o	o	
	8. Strapping an ankle	o	o	o	o	
	9. Cryotherapy (warts)	o	o	o	o	
	10. Setting up an intravenous infusion	o	o	o	o	
52	When do you, or your staff, measure blood pressure? (more than one answer possible)	☐ In connection with relevant clinical conditions				
		☐ On request				
		☐ Routinely in office contacts with adults (regardless of the reason for visit)				

		☐In adults invited for this purpose			

53	When do you, or your staff, measure blood cholesterol level? (More than one answer possible)	☐In connection with relevant clinical conditions			
		☐On request			
		☐Routinely in office contacts with adults (regardless of the reason for visit)			
		☐In adults invited for this purpose			
		☐No such measures			
54	To what extent are you involved in the health education as regards the following topics: (More than one answer possible)	**Not involved**	**In connection with normal patient contacts (During one to one consultation)**	**In group sessions or special programmes**	
	1.Smoking	☐	☐	☐	
	2. Diet	☐	☐	☐	
	3. Problematic use of alcohol	☐	☐	☐	
	4. Physical exercise	☐	☐	☐	

55	Are you or your practice staff involved in the following activities:	**Involved**	**Not Involved**	
	1. Routine antenatal care	o	o	
	2. Immunisation of children (as part of a programme)	o	o	
	3. Paediatric surveillance of children under 4 years	o	o	i.e. child development assessment
	4. Influenza vaccination (as part of a programme)	o	o	e.g vaccination prior to Haj

	5. Palliative care	○	○				
56	During the past 12 months, have you offered (a) special session(s) or clinics for the following groups?	**Yes**	**No**				
	1. Diabetic patients	○	○				
	2. Hypertensive patients	○	○				
	3. Pregnant women	○	○				
	4. Elderly	○	○				
57	If you were confronted through your patient contacts with the following occurrences, would you report this (for instance to an authority)?	**Yes**	**Probably Yes**	**Probably No**	**No**	**Don't Know**	
	1. Repeated accidents in an industrial setting	○	○	○	○	○	Industrial accident reporting form available in KK
	2. Frequent respiratory problems in patients living near a certain industry	○	○	○	○	○	
	3. Repeated cases of food poisoning among people living in a certain district	○	○	○	○	○	Filling up the Notifiable disease form.
58	In the past 12 months, about how many days/weeks altogether have you been away from the practice due to:						
	1. Attending conferences or other educational activities	____days/weeks					
	2. Research activities	____days/weeks					
	3. Vacations	____days/weeks					
	4. Illness	____days/weeks					
59	To what extent do you agree with the following statements?	**Strongly Agree**	**Agree**	**Disagree**	**Strongly Disagree**		
	1. I feel that some parts of my work do not really make sense	○	○	○	○		
	2. My work still interests me as much as it ever did	○	○	○	○		
	3. My work is overloaded with unnecessary administrative detail	○	○	○	○		
	4. I have too much stress in my	○	○	○	○		

	current job					
	5. Being a medical doctor is a well respected job	○	○	○	○	
	6. In my work there is a good balance between effort and reward	○	○	○	○	
60	What is your qualification?	○ Medical officer/GP				
		○ Family Medicine specialist				
		○ Medical officer in charge				
		○ Other specialist				

Time End:
